# First Trimester Circulating MicroRNA Biomarkers Predictive of Subsequent Preterm Delivery and Cervical Shortening

**DOI:** 10.1038/s41598-019-42166-1

**Published:** 2019-04-10

**Authors:** Joanna Cook, Phillip R. Bennett, Sung Hye Kim, Tiong Ghee Teoh, Lynne Sykes, Lindsay M. Kindinger, Alice Garrett, Reem Binkhamis, David A. MacIntyre, Vasso Terzidou

**Affiliations:** 1 Parturition Research Group, Institute of Reproductive and Developmental Biology, Imperial College London, Du Cane Road, London, W12 0NN UK; 2 Queen Charlotte’s and Chelsea Hospital, Imperial College Healthcare NHS Trust, Du Cane Road, London, W12 0HS UK; 3 Academic Department of Obstetrics and Gynaecology, Chelsea and Westminster Hospital, 369 Fulham Road, London, SW10 9NH UK; 4 St. Mary’s Hospital, Imperial College Healthcare NHS Trust, Praed Street, London, W2 1NY UK

## Abstract

Preterm birth (PTB) is the leading cause of infant death and disability worldwide. The onset of preterm uterine contractions is preceded by asymptomatic cervical remodelling and ripening, which can be seen on trans-vaginal ultrasound as cervical shortening. This study aimed to identify plasma miRNA biomarkers that predict preterm birth and/or cervical shortening. We collected serial plasma samples from pregnant women prospectively from 12 to 22 weeks gestation. The nCounter miRNA assay was used to identify differentially expressed miRNAs associated with spontaneous PTB and/or cervical shortening (n = 16 term no short, n = 13 preterm, n = 24 short). Predictive values of the miRNA biomarkers were confirmed in an independent validation cohort consisting of 96 women who delivered at term, 14 preterm and 21 early cervical shortening at <20 weeks gestation. Nine miRNAs (hsa-let-7a-5p, hsa-miR-374a-5p, hsa-miR-15b-5p, hsa-miR-19b-3p, hsa-miR-23a-3p, hsa-miR-93-5p, hsa-miR-150-5p, hsa-miR-185-5p and hsa-miR-191-5p) were differentially expressed (*P* < 0.001) in women subsequently experiencing PTB or cervical shortening. Hsa-miR-150-5p had the strongest ability to predict PTB (AUC = 0.8725) and cervical shortening (AUC = 0.8514). Plasma miRNAs in the first trimester can predict PTB and cervical shortening in women at risk of preterm delivery. This is a key period in pregnancy when early identification of PTB risk allows time to deliver outcome-modifying interventions.

## Introduction

Preterm birth (PTB) before 37 weeks of gestation is a complex clinical syndrome with multiple aetiologies^1^. It is the leading cause of mortality in children under five years-old worldwide, accounting for more than 1 million deaths per year^2^. The onset of labour, both term and preterm, is diagnosed clinically by uterine contractions, but is preceded by cervical remodelling which occurs over many weeks^3^. Cervical ripening is associated with an inflammatory signature, including up-regulation of prostaglandins, chemokines and cytokines, inflammatory cell infiltration and increased matrix metalloproteinase activity^4^.

Cervical remodelling can be observed on trans-vaginal ultrasound (TVUS) as shortening of the cervix. Shortened cervical length (CL) is a risk factor for spontaneous preterm labour (PTL) in both low- and high-risk pregnancies^5^. It has been reported that ultrasound measurement of CL at 19–24 weeks detects most PTL <28 weeks and 50% of PTL <37 weeks gestation^5^. This is clinically useful in populations already identified as being at high risk of PTL, since interventions (cervical cerclage or progesterone) can reduce the risk of PTB. However, CL measurements using TVUS is not routinely offered to low-risk pregnancies where 60% of all low-risk pregnancies with short cervix at 22–24 weeks deliver <28 weeks of gestation and 90% <32 weeks^6^. It has been suggested that universal CL measurement should be introduced into maternity care for all women for the prediction of PTL^7,8^. However, this would have substantial cost implications, and would require expertise in ultrasound, and multiple appointments. An easily measured circulating biomarker which, when measured early in pregnancy, predicts cervical shortening and PTB, would have significant practical and economic advantages. Early risk identification would also allow earlier intervention, which may be more effective at prolonging pregnancy and improving neonatal outcome^9^.

MicroRNAs (miRNAs) are small, single-stranded, non-coding, 19–25 nucleotide molecules regulating mRNA stability and transcription. They are important regulators of gene expression in almost all eukaryotes; up to 80% of all human genes are thought to be regulated by miRNAs^10,11^. Individual tissues, cells and body fluids have their own unique miRNA expression profiles and these change in pathological processes and disease states. MiRNAs are shed into the circulation where they remain stable and easily measurable, and have thus emerged as potential biomarkers in a variety of diseases including malignancy, infection and inflammatory conditions^12,13^.

Recent studies suggest that specific miRNA expression profiles measured in the cervix or in cells taken from the cervix have the potential to differentiate women destined to deliver preterm^14,15^. However, sampling from the cervix is impractical for obstetric population screening. We hypothesised that, in women at risk of PTB, miRNAs may be shed from reproductive tissues, or other body sites, into the peripheral circulation where they may be used as peripherally available biomarkers of cervical ripening and PTB. To identify miRNA biomarkers of PTB and cervical shortening, we compared circulating miRNA profiles between women at high risk of PTB who delivered preterm with or without cervical shortening in the late first and second trimester of pregnancy with the profiles of women who had no cervical shortening and who delivered at term.

## Results

### Patient characteristics and clinical outcomes

A total of 511 women were recruited and the study was completed in two phases. Firstly, a ‘discovery’ cohort was selected from the first 280 women recruited. Secondly, a ‘validation’ cohort was identified from the subsequent 231 women recruited. In the discovery cohort, the PTB rate before 34 weeks’ gestation was 5.2% (11.9% <37 weeks) and in the validation cohort was 5.3% (12% <37 weeks). In the discovery cohort 13 women delivered preterm (<34 weeks) with complete serial datasets (Table 1). All of these 13 women demonstrated cervical shortening (CL <25 mm prior to 22 weeks) and were defined as ‘OUTCOME 2/PRETERM’. There were no women in the discovery cohort who delivered <34 weeks without cervical shortening. We also identified a further 11 women (3.9%) who demonstrated cervical shortening but delivered at term. These cases were combined with the 13 preterm deliveries to form an outcome group defined as ‘OUTCOME 3/SHORT’. We then identified 16 women who had no cervical shortening and delivered at term to form the control group ‘OUTCOME 1/TERM’.

**Table 1:** Patient Characteristics of Study Cohort.

n Women	Discovery Cohort (n = 40)					Validation Cohort (n = 124)				
	OUTCOME1/TERM	OUTCOME2/PRETERM		OUTCOME3/SHORT		OUTCOME1/TERM	OUTCOME2/PRETERM		OUTCOME3/SHORT	
	16	13		24		96	14		21	
		Short	13	Preterm	13		Short	7	Preterm	10
		No short	0	Term	11		No short	7	Term	11
Age (mean)	35.9	36.6		36.4		34.4	37		35	
Previous PTL/Late Loss (%)	57	67		70		30	85		82	
Previous CT (%)	43	33		41		78	25		35	
Female fetus (%)	56	50		47		52	49		51	
Cervical length TPA (mm)	33	29.1		30.5		32	31		26	
Caucasian (%)	50	32		38		69	70		68	
African-Black (%)	31	53		46		23	30		27	
Asican (%)	19	15		16		8	0		5	

From the validation cohort we selected 124 pregnant women with samples available at time points A (12–14^+6^ weeks) and C (18–21^+6^ weeks). Of these women 96 did not demonstrate cervical shortening and delivered at term (OUTCOME 1/TERM), 14 women delivered preterm (<34 weeks) (OUTCOME 2/PRETERM) and 21 women demonstrated cervical shortening (OUTCOME 3/SHORT). In this cohort, there were 7 women who delivered preterm but did not demonstrate early cervical shortening. Of the 21 women with cervical shortening, 11 women delivered at term and 10 delivered preterm (<34 weeks gestation). Clinical characteristics of the discovery and validation cohort patients are presented in Table 1.

### Identification of circulating maternal plasma mirnas

A total of 56 miRNAs were expressed in more than half of the samples from OUTCOME 1/TERM women at any collection time point (Supplementary Table S1). These circulating miRNA profiles were analysed with principal component analysis (PCA) and partial least squares-discriminant analysis (PLS-DA) where no clear clustering was observed (Supplementary Fig. S1a,b). PCA of miRNA profiles demonstrated patient specific clustering of samples, suggesting that the majority of variation in the miRNA profile from 12 to 21^+6^ weeks gestation is due to patient-patient variability, not gestational age (Supplementary Fig. S1c). These findings were validated using RT-qPCR in an independent group of samples (Supplementary Fig. S1d–f).

### Identification of mirnas predictive of subsequent PTB <34 weeks gestation and/or subsequent cervical shortening

**Figure 1:**
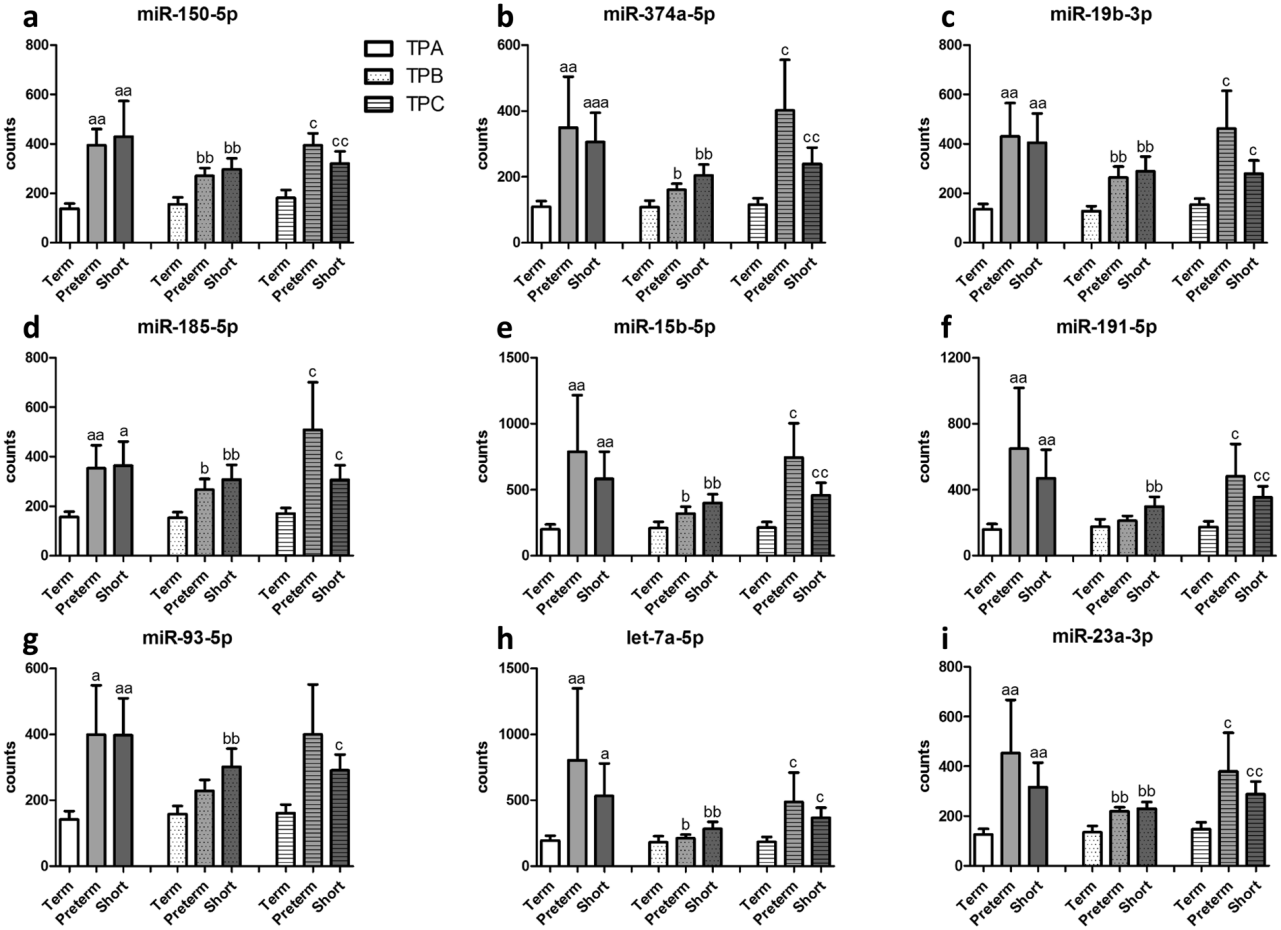
Digital counts for nine cell-free plasma microRNAs with differential expression. Nanostring nCounter miRNA expression profiling assay digital counts for nine specific miRNAs (hsa-miR-150-5p (**a**), hsa-miR-374a-5p (**b**), hsa-miR-19b-3p (**c**), hsa-miR-185-5p (**d**), hsa-miR-15b-5p (**e**), hsa-miR-191-5p (**f**), hsa-miR-93-5p (**g**), hsa-let-7a-5p (**h**), and hsa-miR-23a-3p (**i**)) with differential expression at each timepoint (TPA; time point A 12–14^+6^ weeks, TPB; time point B 15–18^+6^ weeks and TPC; time point C 18–21^+6^ weeks). All data are presented as average and SEM. (a *P* < 0.05, aa *P* < 0.01 vs Term at TPA; b *P* < 0.05, bb *P* < 0.01 vs Term at TPB; c *P* < 0.05, cc *P* < 0.01 vs Term at TPC; Mann-Whitney U test).

A total of 51 circulating miRNAs were expressed above background level in more than 50% of samples in any one of the outcome groups (Table 2). Comparison of the expression profiles of these 51 miRNAs in the OUTCOME 1/TERM group with the OUTCOME 2/PRETERM and OUTCOME 3/SHORT groups from the discovery cohort led to the identification of nine differentially expressed miRNAs (*P* < 0.05), all of which significantly differed in at least one of the three time points (Fig. 1). These differences were maintained following technical replication by RT-qPCR-based assays (Table 3).

**Table 2:** MicroRNAs expressed in human plasma above background level.

Plasma microRNAs expressed above background levels		
hsa-let-7a-5p	hsa-miR-150-5p	hsa-miR-26b-5p
hsa-let-7b-5p	hsa-miR-15a-5p	hsa-miR-29b-3p
hsa-let-7d-5p	hsa-miR-15b-5p	hsa-miR-302d-3p
hsa-let-7g-5p	hsa-miR-16-5p	hsa-miR-30e-5p
hsa-let-7i-5p	hsa-miR-181a-5p	hsa-miR-320e
hsa-miR-106a-5p+hsa-miR-17-5p	hsa-miR-185-5p	hsa-miR-342-3p
hsa-miR-106b-5p	hsa-miR-188-5p	hsa-miR-374a-5p
hsa-miR-107	hsa-iR-191-5p	hsa-miR-378e
hsa-miR-1183	hsa-miR-199a-3p+hsa-miR-199b-3p	hsa-miR-4454
hsa-miR-122-5p	hsa-miR-19b-3p	hsa-miR-451a
hsa-miR-125b-5p	hsa-miR-20a-5p+hsa-miR-20b-5p	hsa-miR-514b-5p
hsa-miR-126-3p	hsa-miR-222-3p	hsa-miR-570-3p
hsa-miR-130a-3p	hsa-miR-223-3p	hsa-miR-574-5p
hsa-miR-142-3p	hsa-miR-22-3p	hsa-miR-598
hsa-miR-144-3p	hsa-miR-23a-3p	hsa-miR-720
hsa-miR-146a-5p	hsa-miR-25-3p	hsa-miR-92a-3p
hsa-miR-148b-3p	hsa-miR-26a-5p	hsa-miR-93-5p

The 51 circulating miRNAs listed were identified by the nCounter miRNA expression profiling assay to be expressed above background level.

**Table 3:** Cell-free plasma microRNAs with differential expression in the discovery cohort.

miRNA	Outcome	TIME POINT A			TIME POINT B			TIME POINT C		
		Mean relative expression	SEM	P value	Mean relative expression	SEM	P value	Mean relative expression	SEM	P value
hsa-miR-150-5p	Outcome 1- TERM	1.0	0.3		1.0	0.2		1.0	0.1	
	Outcome 2- PRETERM	6.2	3.1	0.04	8.8	3.9	0.05	15.4	5.7	0.005
	Outcome 3- SHORT	4.5	1.7	0.05	6.2	2.0	0.02	9.3	2.6	0.005
hsa-miR-374a-5p	Outcome 1- TERM	1.0	0.4		1.0	0.2		1.0	0.3	
	Outcome 2- PRETERM	6.2	3.7	0.04	8.2	4.3	0.03	11.4	7.0	0.05
	Outcome 3- SHORT	5.8	2.4	0.04	9.5	3.6	0.009	8.6	3.9	0.03
hsa-miR-19b-3p	Outcome 1- TERM	1.0	0.3		1.0	0.2		1.0	0.2	
	Outcome 2- PRETERM	4.1	1.6	0.03	4.5	1.6	0.03	8.0	4.2	0.05
	Outcome 3- SHORT	6.1	3.1	0.01	5.7	1.5	0.004	6.2	2.3	0.08
hsa-miR-185-5p	Outcome 1- TERM	1.0	0.2		1.0	0.3		1.0	0.2	
	Outcome 2- PRETERM	6.2	2.7	0.03	4.5	1.5	0.08	8.8	1.9	0.002
	Outcome 3- SHORT	4.9	1.5	0.05	6.8	2.9	0.04	5.6	1.3	0.01
hsa-miR-15b-5p	Outcome 1- TERM	1.0	0.3		1.0	0.2		1.0	0.2	
	Outcome 2- PRETERM	11.3	8.8	0.03	8.1	4.2	0.04	17.4	6.1	0.04
	Outcome 3- SHORT	7.5	4.4	ns	8.0	3.0	0.02	11.4	3.1	0.02
hsa-miR-191-5p	Outcome 1- TERM	1.0	0.4		1.0	0.3		1.0	0.1	
	Outcome 2- PRETERM	5.5	5.2	ns	7.0	4.0	0.04	6.6	5.3	0.04
	Outcome 3- SHORT	4.0	2.8	ns	9.2	4.5	ns	10.7	2.6	0.04
hsa-miR-93-5p	Outcome 1- TERM	1.0	0.2		1.0	0.1		1.0	0.2	
	Outcome 2- PRETERM	4.5	2.6	ns	5.3	2.2	ns	6.6	2.6	0.02
	Outcome 3- SHORT	3.9	1.3	ns	5.1	1.2	0.004	5.1	1.6	0.05
hsa-let-7a-5p	Outcome 1- TERM	1.0	0.2		1.0	0.1		1.0	0.1	
	Outcome 2- PRETERM	6.8	4.7	ns	10.5	4.9	0.06	24.2	8.1	0.004
	Outcome 3- SHORT	5.4	2.4	ns	8.6	2.7	ns	14.3	3.6	0.01
hsa-miR-23a-3p	Outcome 1- TERM	1.0	0.2		1.0	0.2		1.0	0.1	
	Outcome 2- PRETERM	14.1	10.1	ns	6.5	2.2	0.004	21.2	9.3	0.02
	Outcome 3- SHORT	8.8	4.9	ns	8.0	3.4	0.02	12.8	4.1	0.04

Expression of specific miRNAs was measured using RT-PCR in women who had normal cervical length and delivered at term (Outcome 1) and was compared to women who delivered preterm (Outcome 2) or women who developed early cervical shortening (Outcome 3). Results are presented as fold change, mean relative expression and IQR at each gestational time-point (time point A:12–14^+6^ weeks, time point B: 15–18^+6^ weeks and time point C :18–21^+6^ weeks).

Validation of the nine miRNA biomarkers was then performed using RT-qPCR in an independent patient cohort at A and C time points (Supplementary Table S2). Seven of the nine miRNAs demonstrated an AUC > 0.75 and four demonstrated an AUC >  0.80 for their ability to predict PTB (Table 4). The best performing miRNA was hsa-miR-150-5p (AUC 0.87 for PTB and 0.85 for cervical shortening). The specificity of hsa-miR-150-5p (when the DR was 100%) was 64% and 61% for PTB and cervical shortening respectively (Table 4 and Supplementary Table S3).

**Table 4:** Prediction of preterm delivery and cervical shortening by cell-free individual plasma microRNA expression.

miRNA	Outcome	TIME POINT A			
		Area under the ROC curve	P value	Specificity 100% Detection Rate	95% CI
hsa-miR-150-5p	Outcome 1- TERM				
	Outcome 2- PRETERM	0.8725	<0.0001	64.29	53.08% to 74.45%
	Outcome 3- SHORT	0.8514	<0.0001	60.71	49.45% to 71.2%
hsa-miR-374a-5p	Outcome 1- TERM				
	Outcome 2- PRETERM	0.8368	<0.0001	40.48	29.9% to 51.75%
	Outcome 3- SHORT	0.7993	<0.0001	32.14	22.36% to 43.22%
hsa-miR-19b-3p	Outcome 1- TERM				
	Outcome 2- PRETERM	0.8319	<0.0001	41.67	31% to 52.94%
	Outcome 3- SHORT	0.7985	<0.0001	16.67	9.422% to 26.38%
hsa-miR-185-5p	Outcome 1- TERM				
	Outcome 2- PRETERM	0.8214	<0.0001	57.14	45.88% to 67.89%
	Outcome 3- SHORT	0.7707	<0.0001	26.19	17.2% to 36.93%
hsa-miR-15b-5p	Outcome 1- TERM				
	Outcome 2- PRETERM	0.7843	0.0002	30.95	21.31% to 41.98%
	Outcome 3- SHORT	0.7716	<0.0001	30.95	21.31% to 41.98%
hsa-miR-191-5p	Outcome 1- TERM				
	Outcome 2- PRETERM	0.7941	0.0001	39.29	28.8% to 50.55%
	Outcome 3- SHORT	0.7763	<0.0001	34.52	24.48% to 45.69%
hsa-miR-93-5p	Outcome 1- TERM				
	Outcome 2- PRETERM	0.7535	0.001	38.1	27.71% to 49.34%
	Outcome 3- SHORT	0.7517	<0.0001	10.71	5.018% to 19.37%
hsa-let-7a-5p	Outcome 1- TERM				
	Outcome 2- PRETERM	0.6541	0.046	4.819	1.329% to 11.88%
	Outcome 3- SHORT	0.7827	<0.0001	14.46	7.7% to 23.89%
hsa-miR-23a-3p	Outcome 1- TERM				
	Outcome 2- PRETERM	0.6716	0.0262	7.143	2.666% to 14.9%
	Outcome 3- SHORT	0.611	0.0795	7.143	2.666% to 14.9%

ROC curves were calculated to determine the sensitivity and specificity of individual plasma miRNAs to predict preterm delivery subsequent cervical shortening at time point A (12–14^+6^) (Outcome 1/Term-women with no cervical shortening, n = 84, Outcome 2/Preterm- women with spontaneous PTL < 34 weeks, n = 17 and Outcome 3/Short- Women with early cervical shortening prior 22 weeks, n = 28.

A combination of the three miRNAs with the highest individual AUCs (hsa-miR-150-5p, hsa-miR-374a-5p, hsa-miR-19b-3p) did not further improve the diagnostic performance (AUC of 0.87 for PTB and AUC 0.85 for cervical shortening). However, a combination of the seven best performing miRNAs produced a ROC curve with an AUC of 0.91 for PTB (Supplementary Fig. S2).

## Discussion

We report the identification of nine circulating miRNAs whose concentrations in early pregnancy predict both PTL before 34 weeks’ gestation and cervical shortening. As the study was conducted in women at increased risk of PTL receiving CL surveillance, it was a prospective sample and metadata collection study with outcomes defined after delivery. Although our primary outcome was PTL before 34 weeks, it was not considered ethical to withhold intervention (i.e. cervical cerclage) in those women who demonstrated cervical shortening. In these cohorts the intervention was cervical cerclage, not progesterone. Since the intervention may have affected the rate of PTB, we also defined premature cervical shortening as an outcome, irrespective of gestational age at delivery. Nevertheless, we have identified a panel of miRNAs, which are highly predictive of both PTL and premature cervical shortening independent of cervical cerclage as an intervention.

Although the nCounter platform enabled plasma profiling of more than 800 miRNAs simultaneously, cost and practicality considerations limited the sample size of the discovery cohort. However, subsequent technical validation and confirmation of our main findings in an independent patient cohort using a different miRNA quantification platform (RT-qPCR) represents a strength of our study. Independent validation was performed using a ratio of controls to pathological cases (7:1) to better represent the rate of preterm birth in this population. In the discovery cohort, prediction of both PTB and premature cervical shortening was equally robust at all three time points. Considering prediction of PTB or premature cervical shortening is clinically desirable as early in pregnancy as possible, biological validation was performed in samples collected at time point A (12–14^+6^ weeks gestation). Test performance was also undertaken at time point C, which coincides with routine visits to health care providers during pregnancy for diagnostic testing.

Early cervical shortening occurs progressively over several weeks prior to the CL crossing the diagnostic threshold (for our study, <25 mm), just as physiological ripening occurs gradually at term. It is thus reasonable to hypothesize that the underlying mechanism of pathological cervical ripening is active many weeks prior to clinical diagnosis. In support of this concept, Elovitz^14^ showed, using cervical cells collected by speculum examination and cytobrush, that miRNA expression profiles can distinguish asymptomatic women at risk of PTB before their delivery. However, these samples were collected at 20–24 and 24–28 weeks gestation, late in pregnancy for intervention and furthermore sampling using speculum and cytobrush is not suitable for wider population screening.

A number of recent pilot studies have been undertaken in an attempt to examine the capacity of circulating miRNAs to predict subsequent preterm birth. However, these studies have been severely limited by a lack of power, failed to undertake validation or included poorly defined clinical outcome groups that limit the application of proposed biomarkers. Recent pilot study^16^ used the nCounter miRNA profiling array to examine maternal plasma miRNAs at 20 weeks’ gestation in extremely small number of samples (7 preterm and 9 term). They found miR-223 increased in the preterm group and miR-302b, miR-1253, miR-548a, miR-548aa, miR-548ak and miR-548n reduced in the plasma of preterm women. Analysis of our discovery cohort for these targets gave us much lower nCounter values for all the miRNAs apart from miR-223 which had comparable counts to our study (Supplementary Fig. S3a–e). These miRNA targets were not further examined in our validation cohort as they did not qualify as top miRNA targets contributing to the separation of our discovery cohort. Another example is a recent examination of 30 miRNAs measured in first trimester peripheral blood mononuclear cells in 7 early (<34 weeks) and 7 late preterm deliveries (34–38 weeks) compared to 25 full term deliveries (38–42 weeks)^17^. However, this study included 3 women who delivered >37 weeks in the late preterm group, 43% of early preterm deliveries had preeclampsia and 57% were women presenting with preterm rupture of membranes^17^. The authors examined these miRNAs by RT-qPCR and quantification was recorded as the PCR Ct. A training and a validation set was used together with a microRNA scoring system to calculate risk for preterm delivery. Three of these miRNAs (miR-181a, miR-148a and miR-671) were differentially expressed at time point A (12–14^+6^ weeks) and are shown in Supplementary Fig. S3f–h. The majority of these miRNAs were not consistently expressed in our discovery cohort and therefore did not qualify as candidate biomarkers for subsequent analysis. Two previous studies examining miRNA profiling in women symptomatic of threatened PTL reported inconsistent findings^18,19^. Elovitz *et al* used microarray to examine serum derived miRNAs and could not differentiate women who delivered early from those whose symptoms settled and delivered at term^18^, whereas Fallen *et al* used a small RNA library construction protocol in circulating plasma from women with symptoms of preterm labour and identified a spectrum of miRNA changes^19^. These studies examined a very different obstetric population which had already developed uterine contractions at later gestational ages (24–29 weeks gestation) for a variety of reasons. The onset of contractions would also likely have a significant effect upon release of miRNAs from within the cervix or uterus into the circulation.

The best performing miRNA detected in our study, hsa-miR-150-5p, is a biologically plausible biomarker of cervical shortening and PTB. It is a conserved miRNA, located on chromosome 19, expressed in the cervix and placenta and regulated by the inflammation associated transcription factor NFkappaB, which we have shown to be pivotal to labour onset^20,21^. Known targets of hsa-miR-150-5p include members of the membrane-type-1 matrix metalloproteinases involved in cervical ripening^22,23^. Consistent with a role in the pathophysiology of PTL, we have found that its plasma concentrations rise from 12–14^+6^ through 15–18^+6^ to 19–21^+6^ weeks gestation (6-fold, 8-fold and 15-fold respectively) in women who deliver preterm and (4.5-fold, 6-fold and 9-fold respectively) in women who demonstrate early cervical shortening. The observed increased detection of plasma hsa-miR-150-5p in women with later cervical shortening/weakness and PTB could be secondary to increased active shedding from the reproductive tissues or alternatively it could be indicative of underlying increased expression. There also exists a class of miRNAs that are preferentially sorted and packaged into exosomes for excretion into the circulation, and hsa-miR-150-5p is an example of this group^24^.

The assessment of circulating miRNAs in a cohort of women at high risk of PTL could be considered both a strength and a weakness of our study. The recruitment of women at high-risk of PTL permitted enrichment of those women most likely to subsequently deliver preterm and/or demonstrated cervical shortening. It also further focused our study towards women with a more defined underlying PTB aetiology; women with abnormal cervical function or vaginal host immune interactions characterised by early cervical shortening. Although our primary outcome was preterm delivery before 34 weeks, it was not considered ethical to withhold intervention (i.e. cervical cerclage) in those women who demonstrated cervical shortening. We therefore consider that comparing two pathology outcomes (preterm delivery before 34 weeks and cervical shortening) with the control group (term delivery without cervical shortening) represent more appropriately this prospective sample and metadata collection study with outcomes defined after delivery. All the women that delivered preterm in our discovery cohort demonstrated preterm cervical shortening but 11 women who demonstrated cervical shortening delivered at term after receiving an USS indicated cervical cerclage. In our validation cohort, seven women delivered preterm without a shortened cervix <25 mm and therefore did not receive intervention but their mean Time Point C cervical length was 27 mm (range 26–29 mm), only slightly above the clinical cut-off value for intervention and close to the 10^th^ percentile for the general population^25^. Therefore, these subjects likely fall within an aetiological group characterised by cervical weakness and increased risk of delivery <34 weeks, and represent those women at highest risk of associated maternal and neonatal infection and/or inflammation. Early gestational age at delivery and infection/inflammation are the major risk factors for prematurity associated cerebral palsy and adverse neurodevelopmental outcomes^26^. This is also a group with high PTB recurrence rates, and increased utilisation of medical resources. Currently, women at increased risk of PTB are likely to be offered more intensive antenatal care and CL surveillance to identify those likely to benefit from interventions; commonly progesterone and/or cervical cerclage. In most clinical settings, women in their first pregnancy are not routinely offered CL measurement, for cost and practicality reasons. Screening in early pregnancy using circulating miRNAs could therefore offer an effective triage of women at risk of PTL allowing intervention at an earlier time in pregnancy and more effective targeting of resources.

The value of miRNA screening for early PTB will be to triage women already defined at increased risk, and to identify women at risk in their first pregnancy. This will require a test with a high sensitivity. Any woman defined through screening at high risk could then undergo TVUS surveillance which although expensive and invasive, is widely accepted and clinically used. Our data suggest that hsa-miR-150-5p provides a ‘cut-off’ or ‘screen-positive’ value that gives 100% sensitivity for both PTL and cervical shortening with 60% specificity meaning that the ratio of ‘screen-positive’ to ‘true positive’ will be less than 2 to 1. Refinement of risk at the end of the first trimester, in women already identified of being at increased risk of cervical shortening and PTL will allow improved targeted surveillance and delivery of interventions at an earlier stage in pregnancy, which may improve effectiveness. We further anticipate that miRNA screening could also be used to identify women at risk of early PTL in otherwise low-risk general populations, although this will require further study.

## Methods

### Participant recruitment and sample collection

Ethical approval was granted by the Hertfordshire Research Ethics Committee (22/02/2011-REC reference number 11/H0311/6) and informed written consent was received from all participants. All research was performed in accordance with the relevant guidelines and regulations. Prospective serial maternal plasma samples and CL measurements at three time-points in mid-pregnancy: 12–14^+6^ (time-point A), 15–17^+6^ (time-point B) and 18–21^+6^ (time-point C) weeks gestation were collected from women with singleton pregnancies attending preterm birth prevention clinics at Imperial College Healthcare NHS Trust hospitals in London, UK. Women were defined as being at increased risk of PTB because of previous mid-trimester loss and/or PTB, or previous excisional treatment for cervical intraepithelial neoplasia (CIN). At each sampling, whole blood was collected and placed immediately on ice before centrifugation at 1300 × *g* for 10 min at 4 °C within 30 min of collection. Isolated plasma was stored in 1000 μl aliquots in RNase-free microtubes at −80 °C. Samples demonstrating macroscopic haemolysis were discarded.

Women who developed obstetric complications (pre-eclampsia, gestational diabetes, fetal growth restriction, obstetric cholestasis) were excluded from the study. Following delivery, subjects were categorised into three different outcome groups. Outcome 1/TERM comprised women with uncomplicated pregnancies and no cervical shortening who delivered at term. Outcome 2/PRETERM comprised women who spontaneously delivered preterm (<34 weeks) and outcome 3/SHORT comprised women who demonstrated second trimester cervical shortening. Cervical shortening was defined as a CL measurement of <25 mm at time-points B or C when measurement at time-point A was >25 mm. All CL measurements were obtained using TVUS. All women who developed cervical shortening underwent cervical cerclage as per local guidelines. Women included in our study had not received progesterone before or after cervical shortening.

### RNA extraction

Plasma aliquots were thawed on ice and centrifuged at 800 × *g* for 10 min at 4 °C to minimise cellular and platelet RNA contamination^27^. The upper 750 μl was removed for processing and the remaining plasma was discarded. RNA was extracted using the ‘Plasma/Serum Circulating and Exosomal RNA Purification Mini Kit (Slurry-Format)’ (Norgen-Biotek, Ontario, Canada). A spike-in of 5000 attomoles of synthetic cel-254 (sequence:UGCAAAUCUUUCGCGACUGUAGG, Integrated DNA Technologies BVBA, Leuven, Belgium) was added following the addition of lysis and denaturing buffers to allow downstream normalisation of any technical variation to the extraction process. Eluted RNA was further purified and concentrated using Amicon Ultra YM-3 columns (Millipore, Darmstadt, Germany) to a final volume of 25 μl.

### Nanostring nCounter plasma miRNA assay and RT-qPCR for miRNA expression and quantification

nCounter miRNA plasma profiling was used to compare plasma miRNA expression across gestational time points in a ‘discovery cohort’ consisting of maternal plasma samples collected serially at three time-points in mid-pregnancy: 12–14^+6^ (time point A), 15–17^+6^ (time point B) and 18–21^+6^ (time point C) weeks gestation. The nCounter^TM^ plasma miRNA cassette (Nanostring, Seattle, USA) permits target miRNA expression levels to be directly assessed, without cDNA synthesis or enzymatic reactions, via two sequence-specific probes^28^.

RT-qPCR was then used to confirm the data derived from the nCounter assay and to measure miRNA expression in an independent validation population cohort. For this, RNA was reverse transcribed to cDNA using miRCURY LNA^TM^ Universal RT miRNA cDNA synthesis kit II (Exiqon, Vedbaek, Denmark), following the addition of 0.625 μl synthetic miRNA UniSp6 (10^8^ copies/μl) (Exiqon, Vedbaek, Denmark) to allow downstream normalisation of any technical variation to the reaction. A constant initial volume of 750 μl plasma was used for each extraction. To minimise the impact of endogenous inhibitors, the reverse transcription (RT) reaction for plasma miRNAs was optimised by comparing Ct values derived from multiple volumes of RNA in-put (0.01 μl, 0.1 μl, 0.5 μl, 1 μl, 2 μl and 3 μl) into 10 μl total RT reaction. 1 μl RNA/10 μl RT reaction was found to be optimal. RT-qPCR was performed on ABI StepOnePlus (LifeTechnologies, Paisley, UK) using custom ‘pick and mix’ PCR panels containing LNA^TM^ (locked nucleic acid) primers for the nine miRNA targets, according to the manufacturer’s instructions (Exiqon, Vedbaek, Denmark). Optimised and experimentally validated miRCURY LNA™ Universal RT microRNA PCR system primer sets were purchased from Exiqon. LNA^TM^ primers were specifically designed to have maximum sensitivity with at least 95% efficiency, which enables multiple miRNA targets to be assayed under the same experimental conditions. Melt curve analysis was used to confirm presence of a single PCR product. Duplicate ‘no template’ control reactions were performed with all assays, confirming the absence of RNA contamination. A calibrator primer was included in all plates which showed that the intra-assay variation (coefficient of variance) of Ct values was <2%.

### Statistical analysis of plasma miRNA expression

The background signal of the nCounter assay was defined as two standard deviations above the mean of negative control probes and subtracted from the raw miRNA molecule counts. Expression counts were normalised to the mean expression of the top 100 expressed miRNAs. Raw values of nCounter expression counts are included as Supplementary Data. MiRNA probes without expression above background in more than half of the samples from any clinical group were removed from further analysis. Samples with very high expression of haemolysis-associated miRNAs, hsa-miR-16-5p, hsa-miR-25-3p and hsa-miR-93-5p were removed from analysis (n = 2)^29^.

We initially compared log-transformed geometric means of expression counts (which assumes unequal variance) between gestational groups, using nSolver v2.0 software (Nanostring Technologies, Seattle, USA). MiRNAs with significant differences in mean expression counts between time points were defined as *P* < 0.05 and a false discovery rate (FDR) <0.05. Datasets were assessed using the D’Agostino and Pearson omnibus normality test and longitudinal data were interrogated using either the repeated measures ANOVA with the Greenhouse-Geisser correction followed by a post test for linear trend in either direction, or a Freidman test followed by Dunn’s multiple comparison test, if data were found to have a Gaussian or non-Gaussian distribution, respectively. MiRNAs with gestation dependent expression were defined as *P* < 0.05.

nCounter assay data were subjected to unsupervised principal component analysis (PCA) and supervised partial least squares-discriminatory analysis (PLS-DA) using SIMCA-P (Soft independent modelling of class analogies-P; v 13.0.2, Umetrics, Umeå, Sweden). Data were scaled to unit variance by dividing each variable by 1/(S*k*), where S*k* represents the standard deviation value of the variable to ensure that all variables in the model retained equal importance. Goodness-of-fit (R^2^Y) and predictive ability (Q^2^Y) parameters were calculated, the latter by a seven-round internal cross-validation of R^2^Y, and used to assess model fit^30,31^.

Cycle threshold (Ct) values for each miRNA were calculated using StepOne v2.3 software (LifeTechnologies, Paisley, UK). All qPCR reactions were performed with a technical replicate. All assays demonstrated no amplification in ‘no template’ control reactions. Ct values were median normalised firstly to an inter-plate calibrator and then to the extraction and reverse transcription spiked-in controls^32,33^. Distributions were assessed for normality and clinical groups compared using either Student’s unpaired t or Mann-Whitney test as appropriate. Fold change was calculated using 2^−DG^ where DG = mean Ct experimental group - mean Ct normal group.

Detection rate (DR) (or sensitivity) and false positive rate (FPR) (or 1-specificity) for both PTB and cervical shortening were calculated using data from time point A combining both the discovery and validation cohorts. ROC curves were constructed and the AUC for prediction of PTB or cervical shortening was calculated.

## Supplementary information

Supplementary Information

Supplementary dataset

## Data Availability

All data generated or analysed during the currently study are available from the corresponding author on reasonable request.
