# Supplementary material for: First Trimester Circulating MicroRNA Biomarkers Predictive of Subsequent Preterm Delivery and Cervical Shortening
Source: Sci Rep. 2019 Apr 10;9:5861. doi: 10.1038/s41598-019-42166-1 (PMC6458157; doi:10.1038/s41598-019-42166-1)
Supplement: Supplementary file 1 — Supplementary Information [file 41598_2019_42166_MOESM1_ESM.pdf]

Supplementary Figures

Manuscript title:

First Trimester Circulating MicroRNA Biomarkers Predictive of Subsequent Preterm Delivery and Cervical Shortening

List of authors:

J Cook[1,2,4], PR Bennett[1,2], SH Kim[1], TG Teoh[1,4], L Sykes[1,2], LM Kindinger[1,2,4], A Garrett[1], R Binkhamis[1], DA MacIntyre[1] and V Terzidou[1, 3, *]

MicroRNAs expressed in plasma of healthy pregnant women			
hsa-let-7a-5p	hsa-miR-149-5p	hsa-miR-23a-3p	hsa-miR-548aa
hsa-let-7b-5p	hsa-miR-150-5p	hsa-miR-25-3p	hsa-miR-548ai
hsa-let-7g-5p	hsa-miR-15a-5p	hsa-miR-2682-5p	hsa-miR-548al
hsa-miR-106b-5p	hsa-miR-15b-5p	hsa-miR-26b-5p	hsa-miR-570-3p
hsa-miR-107	hsa-miR-16-5p	hsa-miR-297	hsa-miR-574-5p
hsa-miR-1183	hsa-miR-185-5p	hsa-miR-302b-3p	hsa-miR-576-5p
hsa-miR-122-5p	hsa-miR-188-5p	hsa-miR-302d-3p	hsa-miR-579
hsa-miR-1238	hsa-miR-191-5p	hsa-miR-371a-3p	hsa-miR-590-5p
hsa-miR-125b-5p	hsa-miR-199a-5p	hsa-miR-378e	hsa-miR-598
hsa-miR-126-3p	hsa-miR-19b-3p	hsa-miR-4443	hsa-miR-612
hsa-miR-141-3p	hsa-miR-219-5p	hsa-miR-4454	hsa-miR-631
hsa-miR-142-3p	hsa-miR-22-3p	hsa-miR-451a	hsa-miR-761
hsa-miR-144-3p	hsa-miR-222-3p	hsa-miR-495	hsa-miR-769-5p
hsa-miR-148b-3p	hsa-miR-223-3p	hsa-miR-514b-5p	hsa-miR-93-5p

Supplementary Table S1. MiRNAs expressed in human plasma in healthy pregnancy from 12-22 weeks gestation. The miRNAs listed were identified by nCounter miRNA expression profiling assay in more than 50% of samples at any time point.

miRNA	Outcome	TIME POINT A			TIME POINT C		
		Mean relative expression	SEM	P value	Mean relative expression	SEM	P value
	Outcome 1- TERM	1.0	0.8		1.0	0.1	
hsa-miR-150-5p	Outcome 2- PRETERM	8.0	7.0	<0.001	4.6	5.7	0.005
	Outcome 3- SHORT	3.0	0.9	0.001	4.9	2.6	0.005
	Outcome 1- TERM	1.0	0.4		1.0	0.4	
hsa-miR-374a-5p	Outcome 2- PRETERM	3.4	3.7	0.005	3.4	3.5	0.05
	Outcome 3- SHORT	2.5	2.4	0.04	2.5	2.2	0.001
	Outcome 1- TERM	1.0	0.4		1.0	0.3	
hsa-miR-19b-3p	Outcome 2- PRETERM	5.1	4.0	0.002	4.8	4.0	0.03
	Outcome 3- SHORT	2.5	1.3	0.01	4.9	1.9	<0.001
	Outcome 1- TERM	1.0	0.8		1.0	0.3	
hsa-miR-185-5p	Outcome 2- PRETERM	2.0	1.5	0.003	5.5	5.0	0.03
	Outcome 3- SHORT	1.5	1.1	0.01	5.5	2.1	<0.001
	Outcome 1- TERM	1.0	0.7		1.0	0.4	
hsa-miR-15b-5p	Outcome 2- PRETERM	7.8	8.4	<0.0001	3.9	5.0	0.05
	Outcome 3- SHORT	2.2	0.9	0.00	4.7	2.4	<0.001
	Outcome 1- TERM	1.0	0.2		1.0	1.2	
hsa-miR-191-5p	Outcome 2- PRETERM	3.3	1.0	0.015	3.9	21.0	ns
	Outcome 3- SHORT	2.2	0.7	0.04	5.0	9.0	<0.0001
	Outcome 1- TERM	1.0	0.3		1.0	0.2	
hsa-miR-93-5p	Outcome 2- PRETERM	2.4	1.4	0.03	4.9	3.5	0.05
	Outcome 3- SHORT	1.6	0.7	ns	5.5	1.9	<0.0001
	Outcome 1- TERM	1.0	0.4		1.0	0.1	
hsa-let-7a-5p	Outcome 2- PRETERM	1.3	0.6	ns	2.2	1.2	ns
	Outcome 3- SHORT	2.4	2.5	ns	3.0	0.9	0.01
	Outcome 1- TERM	1.0	0.9		1.0	0.6	
hsa-miR-23a-3p	Outcome 2- PRETERM	3.5	4.9	ns	4.2	6.0	0.04
	Outcome 3- SHORT	2.6	2.2	0.01	6.4	3.8	<0.0001

Supplementary Table S2. Cell-free plasma microRNAs with differential expression in the validation cohort. Expression of specific miRNAs was measured using RT-PCR in women who had normal cervical length and delivered at term (Outcome 1) and was compared to women who delivered preterm (Outcome 2) or women who developed early cervical shortening (Outcome 3). Expression was compared at each gestational time-point. Differential expression was defined as a false discovery rate <0.05 in samples taken at time point A ($12\text{-}14^{+6}$ weeks) and/or time point C ($18\text{-}21^{+6}$ weeks) .

miRNA	Outcome	TIME POINT C<br>Area under the ROC curve	P value	Specificity %<br>for 100% Detection Rate	95% CI
	Outcome 1- TERM				
hsa-miR-150-5p	Outcome 2- PRETERM	0.87	<0.0001	62.67	50.73% to 73.57%
	Outcome 3- SHORT	0.84	<0.0001	14.67	7.556% to 24.73%
	Outcome 1- TERM				
hsa-miR-374a-5p	Outcome 2- PRETERM	0.81	0.0006	28	18.24% to 39.56%
	Outcome 3- SHORT	0.83	<0.0001	6.667	2.2% to 14.88%
	Outcome 1- TERM				
hsa-miR-19b-3p	Outcome 2- PRETERM	0.79	0.0013	44	32.55% to 55.94%
	Outcome 3- SHORT	0.79	<0.0001	10.67	4.719% to 19.94%
	Outcome 1- TERM				
hsa-miR-185-5p	Outcome 2- PRETERM	0.76	0.0034	22.67	13.79% to 33.79%
	Outcome 3- SHORT	0.80	<0.0001	22.67	13.79% to 33.79%
	Outcome 1- TERM				
hsa-miR-15b-5p	Outcome 2- PRETERM	0.76	0.0035	17.33	9.565% to 27.81%
	Outcome 3- SHORT	0.77	<0.0001	2.667	0.3246% to 9.303%
	Outcome 1- TERM				
hsa-miR-191-5p	Outcome 2- PRETERM	0.69	0.0375	12	5.637% to 21.56%
	Outcome 3- SHORT	0.66	0.0085	2.667	0.3246% to 9.303%
	Outcome 1- TERM				
hsa-miR-93-5p	Outcome 2- PRETERM	0.75	0.0061	32	21.69% to 43.78%
	Outcome 3- SHORT	0.79	<0.0001	12	5.637% to 21.56%
	Outcome 1- TERM				
hsa-let-7a-5p	Outcome 2- PRETERM	0.69	0.0332	25.33	15.99% to 36.7%
	Outcome 3- SHORT	0.73	0.0002	4	0.8326% to 11.25%
	Outcome 1- TERM				
hsa-miR-23a-3p	Outcome 2- PRETERM	0.81	0.0006	37.33	26.43% to 49.27%
	Outcome 3- SHORT	0.81	<0.0001	17.33	9.565% to 27.81%

Supplementary Table S3. Prediction of preterm delivery and cervical shortening by cell-free individual plasma microRNA expression. ROC curves were calculated to determine the sensitivity and specificity of individual plasma miRNAs to predict preterm delivery or cervical shortening at time point C (18-21^{+6} weeks) (Outcome 1/Term-women with no cervical shortening ,n=75, Outcome 2/ Preterm- women with spontaneous PTL<34 weeks, n=12 and Outcome 3/Short- Women with early cervical shortening prior 22 weeks, n=30).

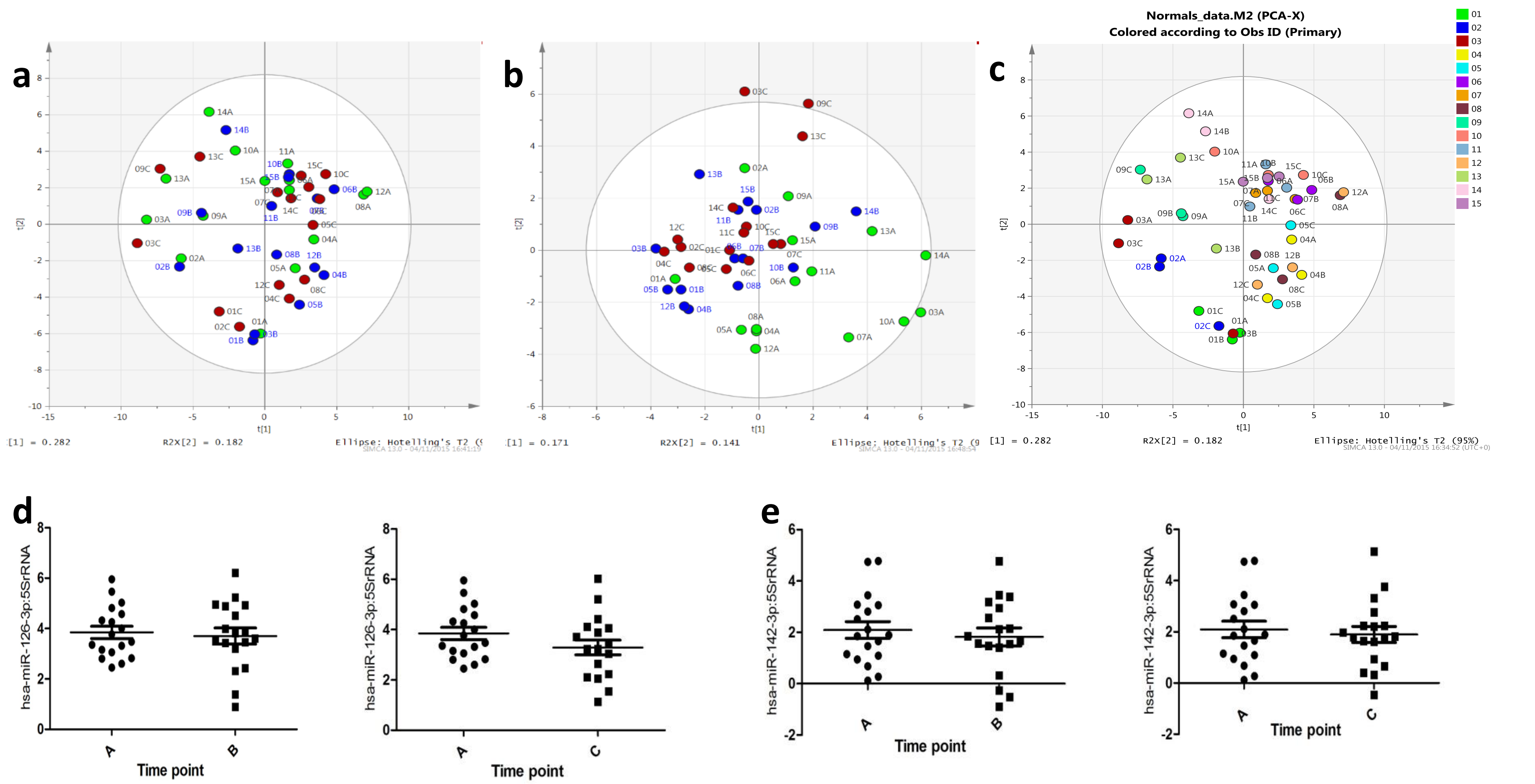

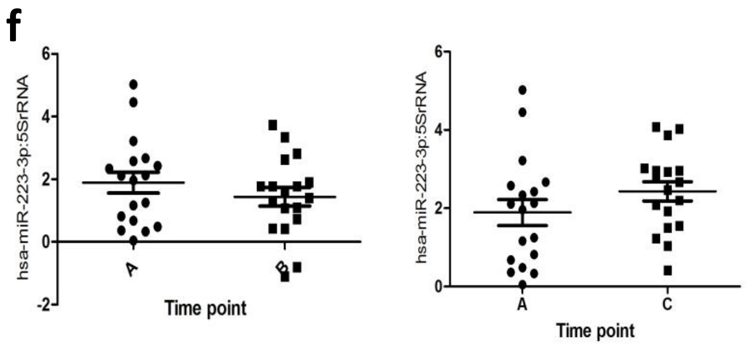

Supplementary Figure S1. Maternal plasma miRNA expression in pregnant women through second trimester. Multivariate modeling with unsupervised principal component analysis identified no clustering in the overall expression profiles of cell-free plasma miRNA suggesting that variation in expression between samples is not attributable to gestational age **(a)**. Furthermore, when data were analysed using supervised partial least squares discriminatory analysis, there was poor separation of gestational groups, indicating that the overall profile of a sample cannot be used to 'predict' gestation **(b)**. However, clustering was observed when expression profiles were assessed by individual woman with principle component analysis, suggesting that the majority of variation in the plasma miRNA profile from 12-21^{+6} weeks gestation is secondary to patient-patient variability **(c)**. Findings were validated using RT-PCR to assess expression of three representative highly expressed miRNAs; hsa-miR-126-3p **(d)**, hsa-miR-142-3p **(e)** and hsa-miR-223-3p **(f)** in an independent group (n=28). The expression of hsa-miR-142-3p, hsa-miR-126-3p and hsa-miR-223-3p did not change with gestation between time points A, B and C.

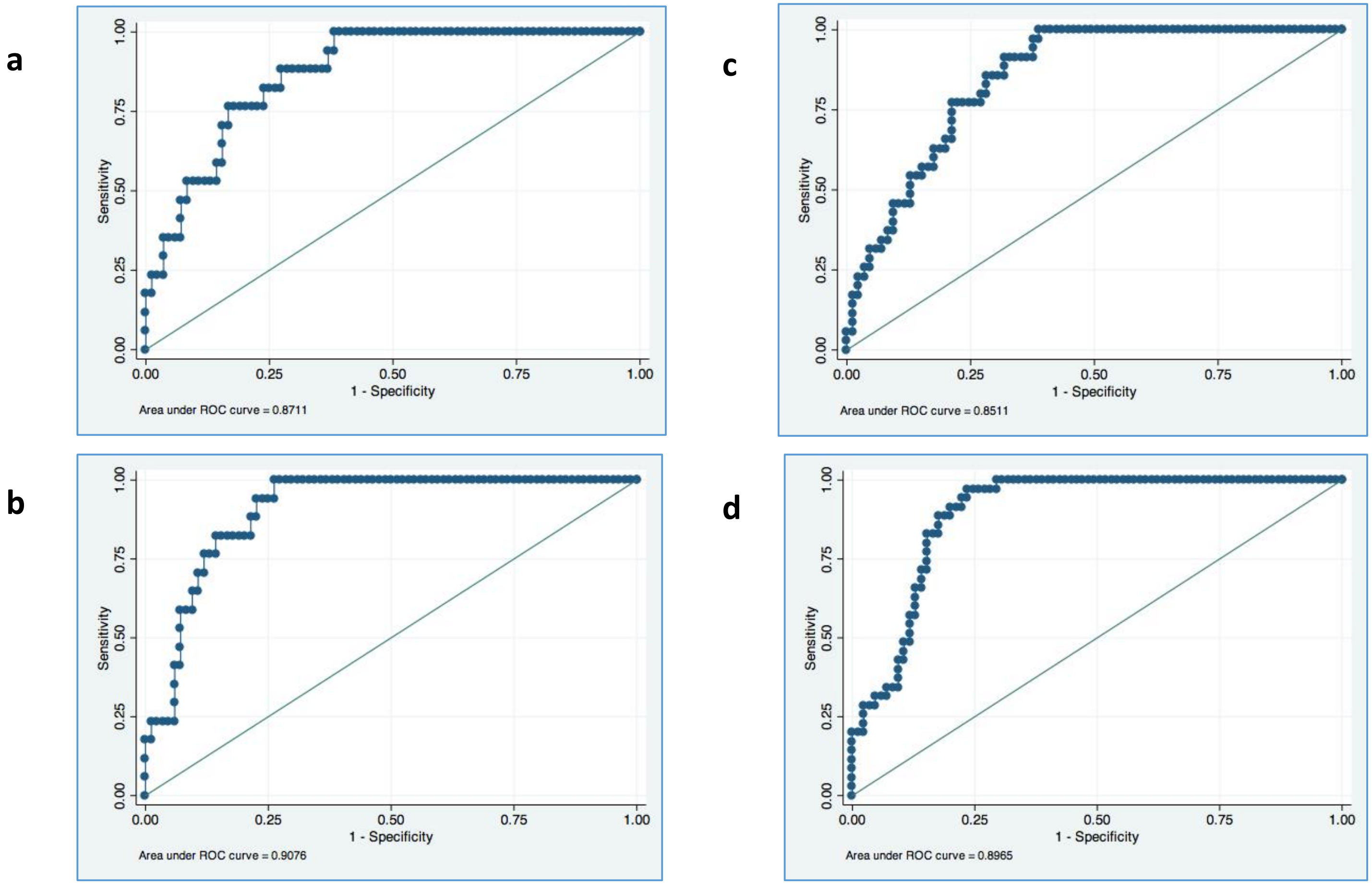

Supplementary Figure S2. Prediction of preterm delivery an cervical shortening by combining individual cell-free plasma microRNA expression at 12-14^{+6} gestation. ROC curves were calculated to determine the sensitivity and specificity by combining **(a)** the top 3 individual miRNAs (AUC=0.8711) **(b)** the top 7 miRNAs to predict preterm delivery (AUC=0.911). **(c)** The top 4 miRNAs to predict cervical shortening gave an AUC of 0.8511 and **(d)** all nine miRNAs AUC=0.8965.

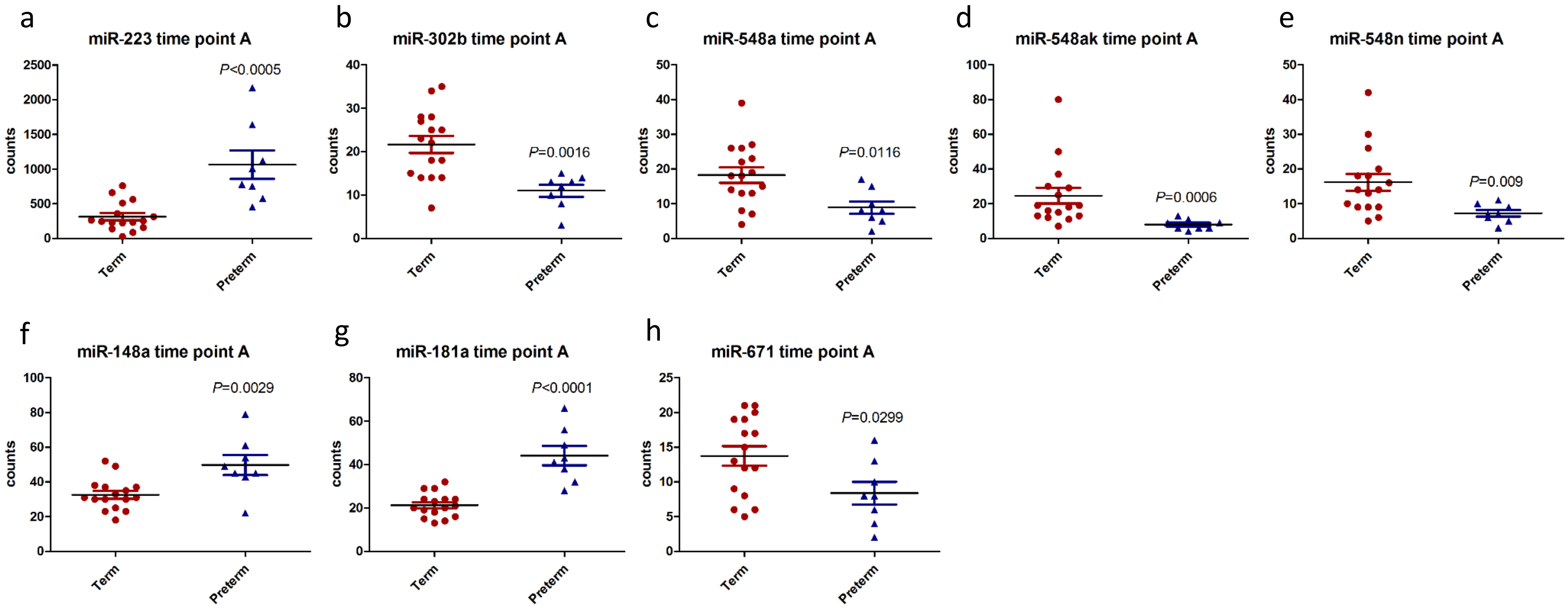

Supplementary Figure S3. Expression of previously reported plasma miRNAs that are differentially expressed in preterm samples at 12-14^{+6} weeks gestation. nCounter miRNA expression profiling assay digital counts of plasma miRNAs, miR at time point A (12-14+6 weeks) from women who went on to deliver at term or preterm. Individual data are presented with SEM. *P* values were obtained by Mann-Whitney U test.
